# Dimethyl 3-(cyclo­propyl­carbon­yl)pyrrolo­[2,1-*a*]isoquinoline-1,2-dicarboxyl­ate

**DOI:** 10.1107/S1600536812015656

**Published:** 2012-04-18

**Authors:** Honglong Xing, Fan Tang, Wei Wang

**Affiliations:** a School of Chemical Engineering, Anhui University of Science and Technology, Huainan 232001, People’s Republic of China; b Key Laboratory for Advanced Technology in Environmental Protection of Jiangsu Province, School of Chemical and Biological Engineering, Yancheng Institute of Technology, Yancheng 224051, People’s Republic of China

## Abstract

In the mol­ecular structure of the title compound, C_20_H_17_NO_5_, two intra­molecular C—H⋯O hydrogen bond generate six- and seven-membered ring motifs. The dihedral angles between the almost planar 13-atom triple-fused-ring system (r.m.s. deviation = 0.003 Å) and the planes of the two meth­oxy­carbonyl substituents are 61.7 (2) and 33.01 (10)°.

## Related literature

For chemical background, see: Michael (2004 ▶); Sriram *et al.* (2005 ▶); Alonso *et al.* (1985 ▶). For the biological activity of indolizine derivatives, see: Shen *et al.* (2010 ▶).
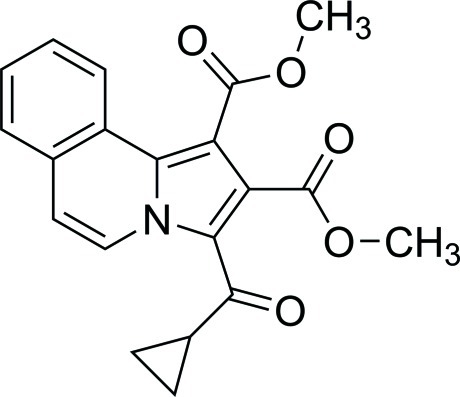


## Experimental

### 

#### Crystal data

C_20_H_17_NO_5_
*M* *_r_* = 351.35
Monoclinic, 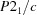

*a* = 7.5910 (15) Å
*b* = 18.436 (4) Å
*c* = 12.162 (2) Å
β = 94.43 (3)°
*V* = 1697.0 (6) Å^3^
*Z* = 4
Mo *K*α radiation
μ = 0.10 mm^−1^
*T* = 293 K
0.30 × 0.20 × 0.10 mm

#### Data collection

Enraf–Nonius CAD-4 diffractometer
Absorption correction: ψ scan (*XCAD4*; Harms & Wocadlo, 1995 ▶) *T*_min_ = 0.971, *T*_max_ = 0.990
3321 measured reflections
3078 independent reflections
2060 reflections with *I* > 2σ(*I*)
*R*_int_ = 0.048
3 standard reflections every 200 reflections intensity decay: 1%

#### Refinement

*R*[*F* ^2^ > 2σ(*F* ^2^)] = 0.056
*wR*(*F* ^2^) = 0.179
*S* = 1.01
3078 reflections
235 parameters
H-atom parameters constrained
Δρ_max_ = 0.30 e Å^−3^
Δρ_min_ = −0.24 e Å^−3^

### 

Data collection: *CAD-4 Software* (Enraf–Nonius, 1989 ▶); cell refinement: *CAD-4 Software*; data reduction: *XCAD4* (Harms & Wocadlo, 1995 ▶); program(s) used to solve structure: *SHELXTL* (Sheldrick, 2008 ▶); program(s) used to refine structure: *SHELXTL*; molecular graphics: *SHELXTL*; software used to prepare material for publication: *SHELXTL* and *PLATON* (Spek, 2009 ▶).

## Supplementary Material

Crystal structure: contains datablock(s) I, global. DOI: 10.1107/S1600536812015656/ff2061sup1.cif

Structure factors: contains datablock(s) I. DOI: 10.1107/S1600536812015656/ff2061Isup2.hkl

Supplementary material file. DOI: 10.1107/S1600536812015656/ff2061Isup3.cml

Additional supplementary materials:  crystallographic information; 3D view; checkCIF report

## Figures and Tables

**Table 1:** Hydrogen-bond geometry (Å, °)

*D*—H⋯*A*	*D*—H	H⋯*A*	*D*⋯*A*	*D*—H⋯*A*
C13—H13*A*⋯O1	0.93	2.27	2.872 (4)	122
C20—H20*A*⋯O4	0.93	2.18	3.019 (4)	150

**Fig. 1.**
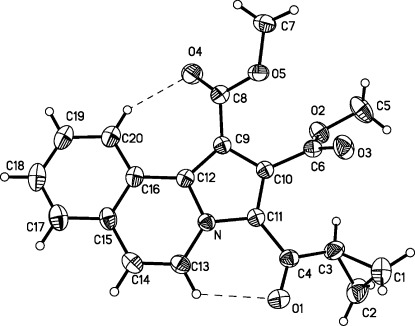
The molecular structure of the title compound, with 30% probability displacement ellipsoids. Dashed lines indicate hydrogen bonds.

## supplementary crystallographic information

### Comment

The indolizine and hydrogenated indolizine structures are found in many alkaloids such as amorine, crythraline, swainsonine, cryptaustoline, cryptowoline (Sriram *et al.* 2005), camptothesin(Michael, 2004), nuevamine (Alonso *et al.*, 1985), *etc*. These natural and many synthetic indolizine derivatives have been found to have a variety of biological activity (Shen *et al.*, 2010).

We report here the synthesis and crystal structure of the title compound (I). The molecular structure of (I) is shown in Fig.1. In the title compound, intramolecular C13 —H13A···O1 and C20—H20A···O4 hydrogen bond generates extra two ring motifs. The dihedral angle between the indolizine ring system and the C20—H20A···O4 plane is 20.40 (4)°.

### Experimental

A mixture of the 2-(2-cyclopropyl-2-oxoethyl)isoquinolinium bromide (10 mmol), acrylonitrile (40 mmol), triethylamine (2 ml) and TPCD (4 g) in DMF (40 ml) was heated at 90° C for 5 h. After cooling, the reaction mixture was poured into an aqueous hydrochloric acid solution (5%, 100 ml), the precipitated crude product was collected by filtration and further purified by silica gel column chromatography with petroleum ether (bp 60–90 °*C*)-ethyl acetate as eluents. Yellow crystal. m.p. 388–389 K, Yield 76%. Single crystals suitable for X-ray diffraction were prepared by slow evaporation of a solution of the title compound in petroleum ether -ethyl acetate(4:1), at room temperature.

### Refinement

The H atoms were fixed geometrically and were treated as riding on their parent C atoms, with C—H distances in the range of 0.93–0.97 Å, and with *U*_iso_(H) = 1.2*U*_eq_(parent atom), or *U*_iso_(H) = 1.5*U*_eq_(C_methyl_).

### Crystal data

**Table:**

C_20_H_17_NO_5_	*F*(000) = 736
*M**_r_* = 351.35	*D*_x_ = 1.375 Mg m^−^^3^
Monoclinic, *P*2_1_/*c*	Melting point: 388 K
Hall symbol: -P 2ybc	Mo *K*α radiation, λ = 0.71073 Å
*a* = 7.5910 (15) Å	Cell parameters from 25 reflections
*b* = 18.436 (4) Å	θ = 9–12°
*c* = 12.162 (2) Å	µ = 0.10 mm^−^^1^
β = 94.43 (3)°	*T* = 293 K
*V* = 1697.0 (6) Å^3^	Block, yellow
*Z* = 4	0.30 × 0.20 × 0.10 mm

### Data collection

**Table:**

Enraf–Nonius CAD-4 diffractometer	2060 reflections with *I* > 2σ(*I*)
Radiation source: fine-focus sealed tube	*R*_int_ = 0.048
Graphite monochromator	θ_max_ = 25.3°, θ_min_ = 2.0°
ω/2θ scans	*h* = 0→9
Absorption correction: ψ scan (*XCAD4*; Harms & Wocadlo, 1995)	*k* = 0→22
*T*_min_ = 0.971, *T*_max_ = 0.990	*l* = −14→14
3321 measured reflections	3 standard reflections every 200 reflections
3078 independent reflections	intensity decay: 1%

### Refinement

**Table:**

Refinement on *F*^2^	Primary atom site location: structure-invariant direct methods
Least-squares matrix: full	Secondary atom site location: difference Fourier map
*R*[*F*^2^ > 2σ(*F*^2^)] = 0.056	Hydrogen site location: inferred from neighbouring sites
*wR*(*F*^2^) = 0.179	H-atom parameters constrained
*S* = 1.01	*w* = 1/[σ^2^(*F*_o_^2^) + (0.1*P*)^2^ + 0.4*P*] where *P* = (*F*_o_^2^ + 2*F*_c_^2^)/3
3078 reflections	(Δ/σ)_max_ < 0.001
235 parameters	Δρ_max_ = 0.30 e Å^−^^3^
0 restraints	Δρ_min_ = −0.24 e Å^−^^3^

### Special details

**Table:**

Geometry. All e.s.d.'s (except the e.s.d. in the dihedral angle between two l.s. planes) are estimated using the full covariance matrix. The cell e.s.d.'s are taken into account individually in the estimation of e.s.d.'s in distances, angles and torsion angles; correlations between e.s.d.'s in cell parameters are only used when they are defined by crystal symmetry. An approximate (isotropic) treatment of cell e.s.d.'s is used for estimating e.s.d.'s involving l.s. planes.
Refinement. Refinement of *F*^2^ against ALL reflections. The weighted *R*-factor *wR* and goodness of fit *S* are based on *F*^2^, conventional *R*-factors *R* are based on *F*, with *F* set to zero for negative *F*^2^. The threshold expression of *F*^2^ > σ(*F*^2^) is used only for calculating *R*-factors(gt) *etc*. and is not relevant to the choice of reflections for refinement. *R*-factors based on *F*^2^ are statistically about twice as large as those based on *F*, and *R*- factors based on ALL data will be even larger.

### Fractional atomic coordinates and isotropic or equivalent isotropic displacement parameters (Å^2^)

**Table:**

	*x*	*y*	*z*	*U*_iso_*/*U*_eq_	
N	0.2043 (3)	0.45752 (11)	0.56131 (17)	0.0407 (5)	
O1	0.0490 (3)	0.31335 (12)	0.57398 (18)	0.0729 (7)	
C1	0.0128 (5)	0.2518 (2)	0.7977 (3)	0.0763 (10)	
H1A	0.0035	0.2474	0.8765	0.092*	
H1B	−0.0987	0.2485	0.7533	0.092*	
O2	0.3127 (2)	0.44915 (12)	0.92593 (15)	0.0583 (6)	
C2	0.1687 (5)	0.22111 (19)	0.7524 (4)	0.0815 (11)	
H2A	0.1537	0.1991	0.6799	0.098*	
H2B	0.2560	0.1979	0.8032	0.098*	
O3	0.0196 (3)	0.44054 (13)	0.88912 (17)	0.0657 (6)	
C3	0.1495 (4)	0.30195 (16)	0.7597 (3)	0.0591 (8)	
H3A	0.2247	0.3260	0.8179	0.071*	
O4	0.4069 (3)	0.64344 (13)	0.76734 (18)	0.0715 (7)	
C4	0.1160 (4)	0.34276 (15)	0.6567 (2)	0.0463 (7)	
O5	0.1973 (3)	0.59404 (11)	0.86128 (17)	0.0613 (6)	
C5	0.2880 (5)	0.4387 (3)	1.0410 (3)	0.0879 (13)	
H5A	0.4009	0.4381	1.0824	0.132*	
H5B	0.2287	0.3934	1.0507	0.132*	
H5C	0.2179	0.4776	1.0667	0.132*	
C6	0.1646 (4)	0.45114 (15)	0.8593 (2)	0.0462 (7)	
C7	0.2330 (5)	0.64687 (18)	0.9471 (3)	0.0694 (10)	
H7A	0.1510	0.6407	1.0026	0.104*	
H7B	0.2206	0.6947	0.9164	0.104*	
H7C	0.3513	0.6405	0.9795	0.104*	
C8	0.3006 (3)	0.59626 (15)	0.7761 (2)	0.0452 (7)	
C9	0.2639 (3)	0.53321 (14)	0.7048 (2)	0.0403 (6)	
C10	0.2004 (3)	0.46707 (14)	0.7437 (2)	0.0403 (6)	
C11	0.1670 (3)	0.41976 (14)	0.6564 (2)	0.0405 (6)	
C12	0.2688 (3)	0.52648 (14)	0.5891 (2)	0.0406 (6)	
C13	0.1793 (3)	0.43333 (16)	0.4534 (2)	0.0472 (7)	
H13A	0.1353	0.3870	0.4387	0.057*	
C14	0.2183 (4)	0.47657 (17)	0.3706 (2)	0.0555 (8)	
H14A	0.1957	0.4607	0.2983	0.067*	
C15	0.2945 (4)	0.54702 (17)	0.3911 (2)	0.0512 (7)	
C16	0.3216 (3)	0.57249 (15)	0.5008 (2)	0.0449 (7)	
C17	0.3413 (4)	0.5916 (2)	0.3040 (3)	0.0644 (9)	
H17A	0.3216	0.5754	0.2317	0.077*	
C18	0.4146 (4)	0.6579 (2)	0.3238 (3)	0.0693 (10)	
H18A	0.4429	0.6871	0.2654	0.083*	
C19	0.4473 (4)	0.68183 (18)	0.4315 (3)	0.0653 (9)	
H19A	0.5020	0.7264	0.4449	0.078*	
C20	0.4002 (4)	0.64068 (16)	0.5189 (3)	0.0553 (8)	
H20A	0.4206	0.6582	0.5905	0.066*	

### Atomic displacement parameters (Å^2^)

**Table:**

	*U*^11^	*U*^22^	*U*^33^	*U*^12^	*U*^13^	*U*^23^
N	0.0414 (12)	0.0421 (12)	0.0390 (12)	0.0036 (10)	0.0048 (9)	−0.0006 (10)
O1	0.0976 (17)	0.0614 (14)	0.0594 (14)	−0.0228 (13)	0.0031 (12)	−0.0076 (11)
C1	0.076 (2)	0.080 (3)	0.075 (2)	−0.0009 (19)	0.0169 (19)	0.0190 (19)
O2	0.0539 (12)	0.0747 (15)	0.0457 (12)	0.0098 (11)	0.0005 (9)	0.0041 (10)
C2	0.101 (3)	0.053 (2)	0.092 (3)	0.0147 (19)	0.022 (2)	0.0156 (19)
O3	0.0523 (12)	0.0861 (16)	0.0604 (13)	−0.0108 (12)	0.0159 (10)	−0.0020 (12)
C3	0.074 (2)	0.0412 (16)	0.061 (2)	−0.0008 (15)	−0.0017 (16)	0.0013 (14)
O4	0.0759 (15)	0.0702 (15)	0.0703 (15)	−0.0316 (13)	0.0186 (12)	−0.0128 (12)
C4	0.0449 (15)	0.0450 (16)	0.0495 (16)	0.0019 (13)	0.0059 (12)	−0.0051 (13)
O5	0.0749 (14)	0.0487 (12)	0.0634 (14)	−0.0122 (10)	0.0248 (11)	−0.0177 (10)
C5	0.092 (3)	0.130 (4)	0.0406 (19)	0.021 (3)	−0.0004 (17)	0.008 (2)
C6	0.0499 (16)	0.0419 (15)	0.0471 (16)	0.0023 (13)	0.0060 (13)	−0.0026 (12)
C7	0.086 (2)	0.062 (2)	0.062 (2)	−0.0102 (18)	0.0194 (18)	−0.0194 (17)
C8	0.0352 (13)	0.0472 (16)	0.0531 (17)	−0.0003 (13)	0.0036 (12)	0.0001 (13)
C9	0.0349 (13)	0.0427 (15)	0.0437 (15)	0.0056 (11)	0.0049 (11)	−0.0003 (12)
C10	0.0379 (13)	0.0417 (15)	0.0413 (15)	0.0045 (11)	0.0035 (11)	0.0006 (11)
C11	0.0373 (13)	0.0423 (15)	0.0421 (15)	0.0050 (11)	0.0038 (11)	0.0011 (12)
C12	0.0339 (13)	0.0402 (14)	0.0479 (16)	0.0071 (11)	0.0039 (11)	0.0020 (12)
C13	0.0448 (15)	0.0531 (17)	0.0428 (16)	0.0060 (13)	−0.0014 (12)	−0.0032 (13)
C14	0.0547 (17)	0.069 (2)	0.0426 (17)	0.0116 (15)	0.0020 (13)	−0.0036 (15)
C15	0.0462 (15)	0.0619 (19)	0.0461 (17)	0.0136 (14)	0.0069 (12)	0.0123 (14)
C16	0.0330 (13)	0.0472 (16)	0.0551 (17)	0.0110 (12)	0.0066 (11)	0.0095 (13)
C17	0.0523 (18)	0.083 (2)	0.059 (2)	0.0119 (17)	0.0109 (14)	0.0175 (18)
C18	0.057 (2)	0.078 (2)	0.075 (2)	0.0110 (18)	0.0161 (17)	0.034 (2)
C19	0.0588 (19)	0.0517 (18)	0.087 (3)	0.0033 (15)	0.0161 (17)	0.0196 (18)
C20	0.0491 (16)	0.0516 (17)	0.066 (2)	0.0040 (14)	0.0112 (14)	0.0080 (15)

### Geometric parameters (Å, º)

**Table:**

N—C13	1.385 (3)	C6—C10	1.483 (4)
N—C12	1.395 (3)	C7—H7A	0.9600
N—C11	1.398 (3)	C7—H7B	0.9600
O1—C4	1.218 (3)	C7—H7C	0.9600
C1—C2	1.458 (5)	C8—C9	1.464 (4)
C1—C3	1.490 (5)	C9—C10	1.406 (4)
C1—H1A	0.9700	C9—C12	1.416 (4)
C1—H1B	0.9700	C10—C11	1.382 (4)
O2—C6	1.334 (3)	C12—C16	1.449 (4)
O2—C5	1.440 (4)	C13—C14	1.335 (4)
C2—C3	1.501 (4)	C13—H13A	0.9300
C2—H2A	0.9700	C14—C15	1.436 (4)
C2—H2B	0.9700	C14—H14A	0.9300
O3—C6	1.201 (3)	C15—C17	1.408 (4)
C3—C4	1.467 (4)	C15—C16	1.414 (4)
C3—H3A	0.9800	C16—C20	1.402 (4)
O4—C8	1.197 (3)	C17—C18	1.356 (5)
C4—C11	1.472 (4)	C17—H17A	0.9300
O5—C8	1.347 (3)	C18—C19	1.387 (5)
O5—C7	1.438 (3)	C18—H18A	0.9300
C5—H5A	0.9600	C19—C20	1.376 (4)
C5—H5B	0.9600	C19—H19A	0.9300
C5—H5C	0.9600	C20—H20A	0.9300
			
C13—N—C12	122.9 (2)	H7B—C7—H7C	109.5
C13—N—C11	127.0 (2)	O4—C8—O5	121.8 (3)
C12—N—C11	110.1 (2)	O4—C8—C9	128.5 (3)
C2—C1—C3	61.2 (2)	O5—C8—C9	109.6 (2)
C2—C1—H1A	117.6	C10—C9—C12	107.1 (2)
C3—C1—H1A	117.6	C10—C9—C8	122.9 (2)
C2—C1—H1B	117.6	C12—C9—C8	129.8 (2)
C3—C1—H1B	117.6	C11—C10—C9	109.7 (2)
H1A—C1—H1B	114.8	C11—C10—C6	124.6 (2)
C6—O2—C5	115.3 (2)	C9—C10—C6	125.7 (2)
C1—C2—C3	60.5 (2)	C10—C11—N	106.5 (2)
C1—C2—H2A	117.7	C10—C11—C4	129.8 (2)
C3—C2—H2A	117.7	N—C11—C4	123.5 (2)
C1—C2—H2B	117.7	N—C12—C9	106.6 (2)
C3—C2—H2B	117.7	N—C12—C16	117.8 (2)
H2A—C2—H2B	114.8	C9—C12—C16	135.7 (3)
C4—C3—C1	120.3 (3)	C14—C13—N	120.0 (3)
C4—C3—C2	118.0 (3)	C14—C13—H13A	120.0
C1—C3—C2	58.3 (2)	N—C13—H13A	120.0
C4—C3—H3A	116.0	C13—C14—C15	121.3 (3)
C1—C3—H3A	116.0	C13—C14—H14A	119.4
C2—C3—H3A	116.0	C15—C14—H14A	119.4
O1—C4—C3	120.7 (3)	C17—C15—C16	119.3 (3)
O1—C4—C11	121.4 (3)	C17—C15—C14	121.3 (3)
C3—C4—C11	117.9 (2)	C16—C15—C14	119.4 (3)
C8—O5—C7	116.7 (2)	C20—C16—C15	118.4 (3)
O2—C5—H5A	109.5	C20—C16—C12	123.1 (3)
O2—C5—H5B	109.5	C15—C16—C12	118.5 (3)
H5A—C5—H5B	109.5	C18—C17—C15	121.1 (3)
O2—C5—H5C	109.5	C18—C17—H17A	119.4
H5A—C5—H5C	109.5	C15—C17—H17A	119.4
H5B—C5—H5C	109.5	C17—C18—C19	119.7 (3)
O3—C6—O2	124.1 (3)	C17—C18—H18A	120.1
O3—C6—C10	123.9 (3)	C19—C18—H18A	120.1
O2—C6—C10	111.9 (2)	C20—C19—C18	121.1 (3)
O5—C7—H7A	109.5	C20—C19—H19A	119.5
O5—C7—H7B	109.5	C18—C19—H19A	119.5
H7A—C7—H7B	109.5	C19—C20—C16	120.4 (3)
O5—C7—H7C	109.5	C19—C20—H20A	119.8
H7A—C7—H7C	109.5	C16—C20—H20A	119.8
			
C2—C1—C3—C4	106.2 (3)	C3—C4—C11—C10	−19.1 (4)
C1—C2—C3—C4	−110.1 (3)	O1—C4—C11—N	−23.9 (4)
C1—C3—C4—O1	−44.0 (4)	C3—C4—C11—N	155.6 (2)
C2—C3—C4—O1	23.8 (5)	C13—N—C12—C9	175.8 (2)
C1—C3—C4—C11	136.5 (3)	C11—N—C12—C9	−2.7 (3)
C2—C3—C4—C11	−155.7 (3)	C13—N—C12—C16	−3.9 (3)
C5—O2—C6—O3	−4.7 (4)	C11—N—C12—C16	177.5 (2)
C5—O2—C6—C10	176.1 (3)	C10—C9—C12—N	1.4 (3)
C7—O5—C8—O4	−6.1 (4)	C8—C9—C12—N	−173.7 (2)
C7—O5—C8—C9	172.0 (3)	C10—C9—C12—C16	−178.9 (3)
O4—C8—C9—C10	150.7 (3)	C8—C9—C12—C16	6.0 (5)
O5—C8—C9—C10	−27.3 (3)	C12—N—C13—C14	0.4 (4)
O4—C8—C9—C12	−34.9 (5)	C11—N—C13—C14	178.7 (2)
O5—C8—C9—C12	147.1 (3)	N—C13—C14—C15	3.1 (4)
C12—C9—C10—C11	0.3 (3)	C13—C14—C15—C17	177.8 (3)
C8—C9—C10—C11	175.9 (2)	C13—C14—C15—C16	−2.9 (4)
C12—C9—C10—C6	−176.5 (2)	C17—C15—C16—C20	−2.2 (4)
C8—C9—C10—C6	−1.0 (4)	C14—C15—C16—C20	178.5 (2)
O3—C6—C10—C11	−63.4 (4)	C17—C15—C16—C12	178.6 (2)
O2—C6—C10—C11	115.8 (3)	C14—C15—C16—C12	−0.8 (4)
O3—C6—C10—C9	113.0 (3)	N—C12—C16—C20	−175.2 (2)
O2—C6—C10—C9	−67.8 (3)	C9—C12—C16—C20	5.1 (4)
C9—C10—C11—N	−2.0 (3)	N—C12—C16—C15	4.0 (3)
C6—C10—C11—N	174.9 (2)	C9—C12—C16—C15	−175.7 (3)
C9—C10—C11—C4	173.5 (2)	C16—C15—C17—C18	1.2 (4)
C6—C10—C11—C4	−9.6 (4)	C14—C15—C17—C18	−179.4 (3)
C13—N—C11—C10	−175.5 (2)	C15—C17—C18—C19	1.2 (5)
C12—N—C11—C10	2.9 (3)	C17—C18—C19—C20	−2.6 (5)
C13—N—C11—C4	8.6 (4)	C18—C19—C20—C16	1.6 (5)
C12—N—C11—C4	−172.9 (2)	C15—C16—C20—C19	0.8 (4)
O1—C4—C11—C10	161.3 (3)	C12—C16—C20—C19	−180.0 (3)

### Hydrogen-bond geometry (Å, º)

**Table:**

*D*—H···*A*	*D*—H	H···*A*	*D*···*A*	*D*—H···*A*
C13—H13*A*···O1	0.93	2.27	2.872 (4)	122
C20—H20*A*···O4	0.93	2.18	3.019 (4)	150
